# Disorders of sex development expose transcriptional autonomy of genetic sex and androgen-programmed hormonal sex in human blood leukocytes

**DOI:** 10.1186/1471-2164-10-292

**Published:** 2009-07-01

**Authors:** Paul-Martin Holterhus, Jan-Hendrik Bebermeier, Ralf Werner, Janos Demeter, Annette Richter-Unruh, Gunnar Cario, Mahesh Appari, Reiner Siebert, Felix Riepe, James D Brooks, Olaf Hiort

**Affiliations:** 1 Department of Pediatrics, Christian Albrechts University of Kiel, Germany; 2 Eppendorf Biochip Systems, Hamburg, Germany; 3 Department of Pediatric and Adolescent Medicine, University of Lübeck, Lübeck, Germany; 4 Department of Biochemistry, Stanford University School of Medicine, CA, USA; 5 Department of Pediatrics, University of Essen, Essen, Germany; 6 Department of Pediatrics, Christian Albrechts University of Kiel, Kiel, Germany; 7 Institute of Human Genetics, Christian Albrechts University of Kiel, Kiel, Germany; 8 Department of Urology, Stanford University School of Medicine, CA, USA

## Abstract

**Background:**

Gender appears to be determined by independent programs controlled by the sex-chromosomes and by androgen-dependent programming during embryonic development. To enable experimental dissection of these components in the human, we performed genome-wide profiling of the transcriptomes of peripheral blood mononuclear cells (PBMC) in patients with rare defined "disorders of sex development" (DSD, e.g., 46, XY-females due to defective androgen biosynthesis) compared to normal 46, XY-males and 46, XX-females.

**Results:**

A discrete set of transcripts was directly correlated with XY or XX genotypes in all individuals independent of male or female phenotype of the external genitalia. However, a significantly larger gene set in the PBMC only reflected the degree of external genital masculinization independent of the sex chromosomes and independent of concurrent post-natal sex steroid hormone levels. Consequently, the architecture of the transcriptional PBMC-"sexes" was either male, female or even "intersex" with a discordant alignment of the DSD individuals' genetic and hormonal sex signatures.

**Conclusion:**

A significant fraction of gene expression differences between males and females in the human appears to have its roots in early embryogenesis and is not only caused by sex chromosomes but also by long-term sex-specific hormonal programming due to presence or absence of androgen during the time of external genital masculinization. Genetic sex and the androgen milieu during embryonic development might therefore independently modulate functional traits, phenotype and diseases associated with male or female gender as well as with DSD conditions.

## Background

Biological sex is the product of genetic and hormonal programs activated during embryonic development, with the most striking gender differences occurring in the internal and external genitalia. Biological sex is a core determinant of sex-specific behavior [1-3] and also a major modifier of a variety of maladies including cancer [4,5] and autoimmune diseases [6]. Delineation of the determinants of biological sex could provide valuable insights into gender-specific behaviors, diseases, treatment responses and outcomes [7]. Sex-specific differences in gene expression have been documented and they are hypothesized to reflect gender-specific development [8,9]. However, sex-specific gene expression appears to be the product of two distinct pathways: sex chromosome genotype (46,XX versus 46,XY) [8,9] and androgen-dependent programming during embryogenesis [10,11]. In contrast to established concepts of androgen programming in rodents [12-14] it has not been possible to tease out the contributions of these two pathways experimentally in normal male and normal female humans since they always work in parallel.

Disorders of sex development (DSD, previously named "intersex") are the product of chromosomal and hormonal abnormalities during development that result in genital ambiguity and incongruent combinations of gender-specific external genitalia, reproductive ducts and gonads in a single individual. Therefore, DSD can serve as an "experiment of nature" to decipher the genetic and hormonal components of sex-specific gene expression in the human. Genetic and hormonal influences on gender extend beyond the reproductive tract, modifying body proportions, hair distribution, gender identity, sex-specific behavior and many other features that also affect DSD patients clinically in multiple ways [1-3,15-17]. Therefore, improved understanding of the architecture of sex specific gene expression may also contribute to better clinical assessment of DSD-patients. In the present study, we hypothesized that sex chromosome controlled gene expression and androgen action during embryonic development in the first trimester as documented by the clinical degree of genital masculinization after birth could be distinguished by comparing normal males and females to individuals with DSD having well-defined clinical, hormonal and molecular characteristics. Because of simple accessibility, lack of overt organ-specific topography biasing gene expression [10,18] and documented androgen receptor expression [19] we used peripheral blood mononuclear cells (PBMC) for genome-wide transcriptome analysis. Our study is the first that profiles blood gene expression data in DSD individuals.

## Results

### Genome-wide transcription profiling

**Figure 1:**
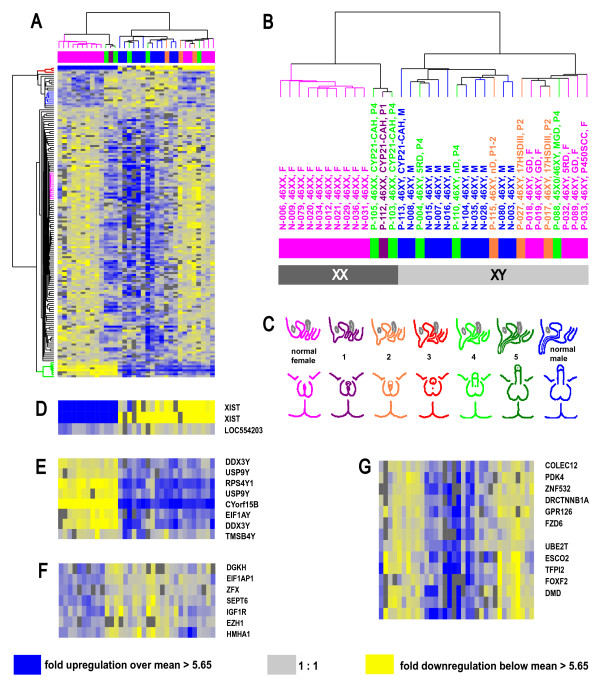
**Hierarchical clustering analysis of 34 microarray experiments on PBMC total RNA derived from 9 normal males, 10 normal females, one 46,XY-male with CAH and 14 individuals with DSD **[see additional files 2, 3, 4, 5]. (A) Transcript levels of 157 transcripts identified by SAM as differing significantly between PBMC from 9 normal males and 10 normal females. Transcripts are grouped by hierarchical cluster analysis and displayed in rows while experiments are displayed in columns. Expression values per gene are centered by the mean log_2 _Cy5/Cy3 normalized ratio across the arrays. Increasing blue intensity in the heat map corresponds to higher relative transcript levels compared to the mean expression level across all 34 array experiments. Increasing yellow intensity corresponds to relatively decreased transcript levels compared to the mean. Dark grey corresponds to missing data. Examples taken from these gene clusters are marked by color within the gene tree on the left of the heat map. The red gene tree corresponds to the enlarged gene cluster D, the blue gene tree corresponds to F, the pink gene tree corresponds to G, and the green gene tree corresponds to E, respectively. Gene symbols of the named transcripts are shown on the right. (B) Enlarged cluster dendrogram of the PBMC samples demonstrating the degree of relatedness (Pearson correlation) between the expression patterns of the 157 transcripts. The length of the arms of the dendrogram reflects the degree of correlation between experiments. Samples are color coded to reflect the degree of external genital virilization according to Prader that had been applied to both XX and XY individuals to enable comparability of genital phenotypes independent of the sex chromosomes. The dark grey bar below the experiment cluster represents individuals with an XX karyotype, light grey represents an XY karyotype. The major subdivison of the individuals corresponds strictly to the karyotype. The second level of subdivision in each of the major arms reflects mostly the phenotype of external genital virilization independent of the karyotype. (C) Schematic representation of the Prader stages of external genital virilization. (D) X-chromosome gene cluster (E) Y-chromosome gene cluster (F) Genes with predominantly higher transcript levels in the phenotypic females and the normal females independent of the karyotype (G) Genes with higher transcript levels in the strongly virilized individuals and the normal males independent of the karyotype.

Transcript profiles for 9 normal male and 10 normal female controls were measured using spotted cDNA microarrays with more than 44,000 cDNA elements representing 26,000 unique genes. The Significance Analysis of Microarrays (SAM) procedure [20] was used to identify 157 sex-specific transcripts (121 unique named genes) (false discovery rate < 0.09): 136 transcripts with higher expression in males (86.6%) and 21 (13.4%) with higher expression in females. Gene expression across these 157 sex-dimorphic transcripts was analyzed across all 19 controls and 14 DSD-patients. The DSD patients were comprised of ten 46,XY subjects whose genitalia ranged from normal female to Prader 4, three 46,XX subjects with masculinized genitalia from high prenatal androgenic steroid levels due to congenital adrenal hyperplasia (CAH, 21-hydroxlase deficiency) and one Prader 4 45,X0/46,XY subject. In addition, one normally masculinized 46,XY male with CAH was investigated (Additional file 1 and Figure 1A, B, C, additional files 2, 3, 4, 5).

Hierarchical cluster analysis sorted the subjects into 2 groups that directly correlated with the sex chromosomes (Figure 1A, B, C). Segregation of the 46,XX and 46,XY subjects into 2 major groups was driven by large differences in expression of 11 of the 157 transcripts corresponding to 8 unique genes (Figure 1B, D, E). High expression of XIST (X (inactive)-specific transcript) and LOC554203 (Xq13.2) characterized genetic females (Figure 1A, D), while DDX3Y (DEAD (Asp-Glu-Ala-Asp) box polypeptide 3, Y-linked) and five additional Y-linked genes characterized genetic males (Figure 1E). RT-PCR confirmed abundant expression of XIST mRNA in two female controls (N-020, N-031) and in two 46,XX virilized CAH-individuals (P-103, P-105) and showed an absence of expression in two 46,XY-controls (N-003, N-015) and two sex-reversed 46,XY DSD-females (P-019, P033). RT-PCR also confirmed that DDX3Y transcript levels were abundant in these same 46,XY individuals, but absent in the 46,XX subjects (data not shown).

**Figure 2:**
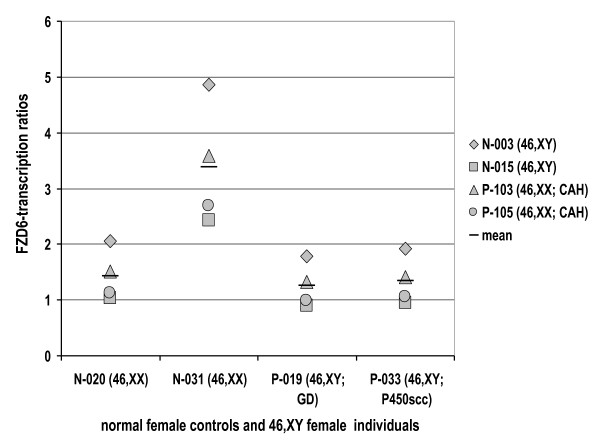
**Ratio of FZD6 transcript levels in 2 46,XY normal males (N-003; N-015) and 46,XX strongly virilized females (P-103; P-105) compared with two 46,XX normal females (N-020; N-031) and two 46,XY females (P-019; P-033) by semi-quantitative RT-PCR**. The y-axis reflects the ratios of expression levels of the normal male – and the strongly virilized DSD individuals, respectively, divided through the phenotypic female individuals (normal females and 46,XY-sex reversed females) as indicated. RT-PCR confirms higher expression of FZD6 in virilized individuals.

Within each of the 46,XX and 46,XY groups were 2 subgroups that largely correlated with the degree of genital masculinization (Figure 1B, F, G). The 46,XX normal female subjects clustered separately from the three 46,XX subjects with genital masculinization and CAH based on differences in expression across 146 of the 157 sex-specific transcripts. Similarly, the 46,XY individuals separated into a group comprised of normal males and highly masculinized individuals with DSD (with one exception) and a group with predominantly female external genitalia, again based on expression differences across the same 146 transcripts. Only two patients did not follow this pattern. Subject P-115, who clustered with phenotypic males, had 46,XY DSD and Prader 1–2 genitalia due to an unclassified defect in androgen biosynthesis. Subject P-088 had mixed gonadal dysgenesis due to 45,X0/46,XY mosaicism and Prader 4 genitalia yet clustered with phenotypic females. Since mosaicism can vary between tissue compartments and since developmental genes may modify the site-specific expression level of the androgen receptor in the external genitalia [21,22], some discrepancies between genital phenotype and systemic androgenization are not unexpected. Using qRT-PCR, we evaluated transcript levels of FZD6 (Frizzled 6), since levels measured by microarray analysis correlated with genital phenotype (Figure 1G). Similar to the microarray experiments, mean FZD6 transcript levels were significantly higher in normal 46,XY-males and in strongly virilized 46,XX-CAH females compared with normal 46,XX-females and 46,XY DSD-females (Figure 2).

### Influence of X- and Y-chromosome genes

**Figure 3:**
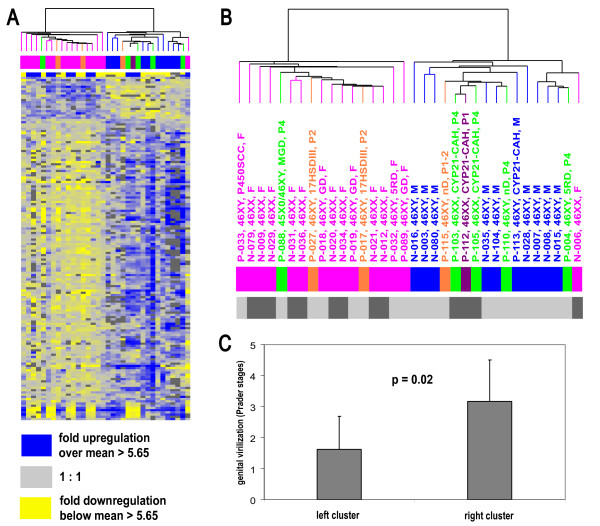
**(A) Hierarchical clustering analysis using the 146 transcripts that did not correlate with karyotype**. Genes of the two sex chromosome gene clusters (see D and E in Figure 1) were excluded from calculating the dendrograms, but are displayed in the figure to show their distribution across the samples. All color codes are the same as in figure 1. (B) Enlarged dendrogram demonstrating that major subdivision of the individuals mirrors the degree of genital virilization independent of karyotype. (C). Mean Prader stage of genital virilization (± 1 SD) of the DSD individuals the main branches of the dendrogram excluding the normal controls. The mean Prader stage on the left side is Prader "1.6" and "3.2" on the right (p = 0.02) [see also additional files 6, 7, 8, 9].

We excluded the 11 X- and Y-linked transcripts that correlated with the two sex chromosome clusters and reanalyzed the samples by hierarchical clustering. With the remaining 146 sex-specific transcripts, subjects no longer grouped according to karyotype, but separated into 2 groups largely distinguished by external genitalia masculinization (Figure 3, additional files 6, 7, 8, 9). Four of the five highly virilized Prader 4 DSD-individuals and all normal males clustered together as did the normal females and subjects with feminized external genitalia. The mean Prader stage of the DSD-individuals excluding the normal controls was 1.6 in the "female" group and 3.2 in those that clustered with the normal males (p = 0.02) (Figure 3). When we excluded all normal controls from the cluster analysis, DSD-individuals separated into the same 2 groups (data not shown) indicating that the normal controls used for SAM had not significantly influenced the clustering of the DSD-patients.

### Influence of concurrent sex hormone levels

At the time of collection of PBMC RNA the 46,XY DSD-subjects with feminized genitalia varied widely in their sex steroid hormone status. Individuals who clustered in the female (left main branch) of Figure 3 included pre- and post-pubertal normal individuals with intact steroid biosynthetic pathways and individuals lacking steroid hormones (subjects P-017, P-032 and P-089 with 46,XY DSD who had been gonadectomized and were not on hormone replacement therapy and subject P-033 who had an inherited defect in, and almost complete lack of, steroid biosynthesis). This cluster also included gonadectomized 46,XY DSD-individuals on estrogen replacement, such as subject P-027 who showed normal adult female plasma estradiol of 492 pmol/L (normal range 73–1065 pmol/L).

Similarly, the phenotypic "male" cluster (right main branch of Figure 3) contained normal postpubertal males (several with documented fertility and presumably adult male testosterone concentrations) and prepubertal individuals (N-028, P-103, P-105, P-110, P-113). The prepubertal subjects P-103, P-105 and P-110 had low serum testosterone levels compared with age – and male sex adjusted normal ranges at the time of blood sampling (P-103: 0.1 nmol/L; P-105: 0 nmol/l; P-110: 0 nmol/l; normal range 0.14–1.32 nmol/l). Subject P-112, who was 46,XX and had a 21-hydroxylase deficiency usually resulting in high levels of androgenic steroids, had been treated prenatally with dexamethasone. Despite having only slightly virilized Prader 1 genitalia, transcript levels of the PBMCs more closely resembled phenotypic males, suggesting residual effects due to elevated adrenal androgens. One subject (P-004), born with Prader 4 external genitalia due to 5α-reductase type II deficiency and gonadectomized in early childhood, was on estrogens and gestagens at the time of blood sampling (plasma estradiol: 822 pmol/L). Therefore, the assignment to either the "female" or "male" clusters based on the 146 sex-specific transcript levels reflected neither pubertal status nor concurrent serum androgen or estrogen levels.

### Biological function of sex-specific transcripts

**Figure 4:**
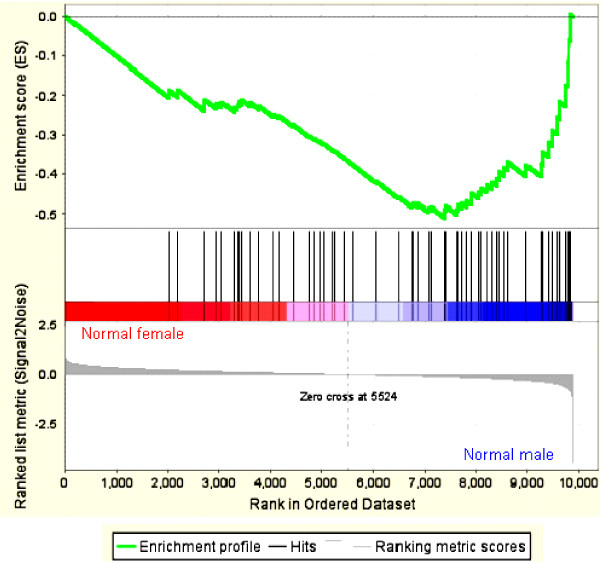
**Gene Set Enrichment Analysis**. Enrichment plot is shown for a set of upregulated genes of a transcriptional profile commonly activated in undifferentiated cancer ("CANCER_UNDIFFERENTIATED_META_UP" as compared to differentiated cancer [22]). The enrichment score (ES, green line) reflects the degree to which the gene set is over-represented at the top or bottom of the ranked list of genes. Black bars illustrate the position of genes belonging to the gene set in the ranked list of genes included in the analysis. The ranked list metric shown in gray measures a gene's correlation with a phenotype. A positive value indicates correlation with "normal female "- phenotype, a negative value with "normal male" – phenotype.

PANTHER (Protein ANalysis THrough Evolutionary Relationships) [23] identified 121 named genes from the 157 sex-specific transcripts. Compared with the NCBI Homo sapiens reference list, 28 biological processes were significantly enriched (p < 0.05) and included developmental processes, extracellular matrix, cell communication and several that are involved in proliferation (table 1). GSEA (Gene Set Enrichment Analysis) [24] was performed using all interpretable genes from normal males and females using 1,107 gene sets from the Molecular Signature Database . 18 gene sets were significantly enriched in males, one in females, of which the majority had been associated with proliferation, often in a context of cancer (e.g., Figure 4[25], ). Interestingly, many of the sex-specific genes identified in our study have published roles in human cancer, including TFPI2 (Tissue factor pathway inhibitor 2) (Figure 1G) [26], LUM (Lumican) [27], PDPN (Podoplanin) [28], SDC1 (Syndecan-1) [29], ESCO2 (Establishment of cohesion 1 homolog 2) (Figure 1G) [30], SSX2IP (Synovial sarcoma, X breakpoint 2 interacting protein) [31], CTGF (Connective tissue growth factor) [32], MMP2 (Matrix metallopeptidase 2) [33]. IGF1R (Figure 1F) [34], SERPINA1 (Serpin peptidase inhibitor, clade A) [35] and SEPT6 (septin 6) (Figure 1F) [36]. A subgroup of these genes function in DNA damage repair, such as CHEK1 (CHK1 checkpoint homolog) [37], XRCC1 (X-ray repair complementing defective repair in Chinese hamster cells 1) [38], H2AFX (H2A histone family, member X) [39], ALKBH (alkylation repair homolog 1) [40] and DCC1 (Defective in sister chromatid cohesion homolog 1) [41].

**Table 1:** *Biological processes *identified by PANTHER [23] as being significantly over-represented (p < 0.05) in the 157-transcript sex-specific SAM-list compared to the NCBI human reference gene list.

**Biological process**	**Detected number of genes in SAM list**	**Expected number of genes based on NCBI reference list**	**p-value**
**Extracellular matrix protein-mediated signaling**	4	0.29	0.000239
**Cell adhesion-mediated signaling**	8	1.8	0.000493
**Cell communication**	15	5.77	0.000674
**Chromatin packaging and remodeling**	6	1.13	0.00101
**Cell cycle**	13	4.8	0.00108
**Mitosis**	7	1.82	0.00246
**Chromosome segregation**	4	0.58	0.0028
**Cell adhesion**	9	2.96	0.00303
**Embryogenesis**	4	0.67	0.0048
**Cell structure**	9	3.27	0.00575
**Gametogenesis**	5	1.15	0.00614
**Cell structure and motility**	12	5.46	0.00878
**Blood clotting**	3	0.44	0.00993
**Developmental processes**	18	10.24	0.0134
**Stress response**	4	0.95	0.0157
**mRNA transcription**	3	9.11	0.0166
**Receptor protein tyrosine kinase signaling pathway**	4	1	0.0187
**Protein modification**	11	5.5	0.0225
**Cell proliferation and differentiation**	10	4.89	0.0253
**DNA metabolism**	5	1.71	0.0294
**Protein metabolism and modification**	22	14.46	0.0294
**Signal transduction**	24	16.21	0.0305
**Lipid, fatty acid and steroid metabolism**	8	3.66	0.0313
**Protein phosphorylation**	7	3.14	0.0389
**Other homeostasis activities**	2	0.33	0.0432
**NO mediated signal transduction**	1	0.05	0.0465
**DNA repair**	3	0.8	0.0474
**Immunity and defense**	11	6.27	0.05

## Discussion

Differences in gene expression that correlate with gender can be dissected into a small set of sex chromosome genes and a larger set of genes that appear to be programmed by androgens, most likely during embryonic development. PBMCs from individuals with diverse well-defined etiologies of DSD including gonadal dysgenesis, defects of androgen biosynthesis, and high levels of androgenic steroids due to 21-hydroxylase deficiency showed strikingly consistent patterns of gene expression across 146 transcripts that correlated most strongly with the genital phenotype. Expression across all 157 transcripts identified as differentially expressed between normal males and females allowed separation of PBMCs into four categories of "PBMC-sexes": chromosomal and phenotypical male, chromosomal and phenotypical female and two intersex constellations in which the chromosomal and steroid influenced transcripts were discordant. While it is possible that transcript profiling of a higher number of DSD-patients could lead to demarcation of even diagnosis-specific patient clusters, the consistency of the transcript profiles across the different diagnoses argues strongly that these four categories are relatively robust.

Animal studies on rodents underlined the importance of sex specificity of the pattern of GH secretion and GH signaling involving STAT5b for maintenance of a large part of sexual dimorphism of gene expression in the liver [42]. Since human PBMC express the GH receptor [43], this mechanism could have a potential influence on the genes detected in the present study. However, in contrast to the extensive sex specific differences in rats with males showing pulsatile GH secretion and females showing more continuous GH levels, data on sex specificity of GH secretion in humans is less clear. While age and puberty status but not sex are important determinants for GH secretion during growth and puberty [44], there is other data showing that GH pulse amplitude but not pulse frequency differ between women and men in adulthood [45]. However, looking at the data of the current study, the correlation between gene expression profiles and genital masculinization was independent of the individuals' sex hormone levels at the time of expression profiling. Puberty status, presence of gonads and sex hormone replacement did not influence expression across the sex-specific transcripts. Additional GSEA on transcription factor targets gene sets did not reveal sex specific enrichment of STAT5b target genes in our study (data not shown). Therefore, we have no experimental evidence that sex specific differences of GH secretion or changes of GH secretion due to age or puberty could have influenced the sex specific gene set of the PBMC of our study. However, it cannot be excluded that a considerably larger set of investigated patients would allow the identification of some sex-dimorphic genes influenced by the GH signaling pathway in PBMCs.

Genital masculinization is a direct consequence of early embryonic androgen action between the 7^th ^and 12^th ^week of gestation. Our findings therefore support the existence of lasting programming of sex-specific genes implemented by presence or absence of androgen during the first trimester of embryogenesis. Sex-specific gene expression patterns have been observed in other tissues [8-11,46-48], yet the relative contribution of the sex chromosomes and androgen programming was unknown. Based on expression patterns readily apparent in PBMCs, cells not usually thought to differ significantly between males and females, our data suggest that many of the sex-specific differences in gene expression are due to the influence of presence or absence of androgens during embryonic development. Given the limited lifespan of PBMCs, the lasting changes in transcript programs due to androgens are likely embedded in progenitor stem cells of fetal leukopoesis. It cannot be excluded that some of the genes detected in our study also played functional roles in androgen programming of lasting PBMC expression patterns during embryogenesis. However, more research is needed to understand the molecular mechanisms of long term hormonal programming, e.g., by studying the effects of androgens on embryonic stem cell differentiation [49]. Whether such programming affects the stem cells of other tissue types is unknown, but highly likely.

In a previous genome-wide assessment of transcript profiles, we have identified transcripts differentially expressed in cultured genital fibroblasts from normal males and 46,XY females with androgen insensitivity syndrome due to inactivating androgen receptor mutations [11]. Similar to the current study we identified a set of transcripts with reproducible differences in gene expression that correlated with the degree of genital masculinization. Since subjects in those studies all had a 46,XY karyotype, the differentially expressed transcripts were almost certainly programmed during development and remained apparent despite serial passage of the fibroblasts in culture. Interestingly, the overlap was restricted to 7 genes. In both datasets FZD6 (Frizzled 6) (Figure 1G), SSX2IP, SDC1, ALKBH, SPRED1 (Sprouty-related, EVH1 domain containing 1), PYCR2 (Pyrroline-5-carboxylate reductase family, member 2) and TOMM40 (Translocase of outer mitochondrial membrane 40) were differentially expressed between the subjects with male and female genitalia. Recent work by Yang and coworkers [9] in which 334 male and female mice were profiled confirms our findings that a significant number of genes differ between males and females. More importantly, as we observed in genital fibroblasts and PBMCs, the degree of overlap of the sex-specific genes between tissues was limited in this study. Our work extends on that of Yang et al by demonstrating that sex steroid induced programming accounts for the majority of the differences in gene expression between phenotypic males and females. Taken together these data suggest that the sex chromosomes to a small degree and androgen signaling during development to a larger degree account for a large part of the differences in gene expression in different tissue types between males and females. Furthermore, the influences of each of these differ significantly between tissue types as evidenced by the limited overlap in the sex-specific transcripts between tissue types. Additional work will be necessary to test whether these differences in gene expression between analogous tissues between males and females underlie developmental, anatomical, and functional differences between the sexes.

Our data also provide a framework for investigating differences in diseases between the sexes and could have future implications for risk assessment and disease monitoring [7]. PBMC expression profiling has been suggested as a diagnostic tool in that germline mutations in disease-causing genes could produce unique transcriptional signatures detectable in blood cells [50-52]. Disease-linked expression profiles are likely to be modified by biological sex [8,9] and might need to be accounted for when developing diagnostic tools. In addition, our data raise the possibility that lasting transcriptional programs that differ between males and females could underlie differential susceptibility to a variety of diseases. For instance, we observed a striking over-representation of genes involved in growth control and cancer in PBMCs between males and females. Whether sex-specific gene expression could affect a tissue's transcriptional background and oncogenic potential or response to anti-cancer therapy is unknown, but merits consideration in light of differences in cancer incidence between the sexes and our new findings.

Our data have relevant implications for understanding the biology of DSD as well as potential future applications in its management. In Western societies, every schoolchild learns that "sex chromosomes" decide "who's a boy and who's a girl". Our data broaden the view of what determines sex and gender by revealing distinct male and female transcript profiles that correlate with either the karyotype or the androgen milieu present during embryonic development. Therefore, our data confirm the concept of long term androgen programming as established in rodent models [12-14] for the first time on the level of the human PBMC-transcriptome. Our data also reveal 2 categories of gene expression in PBMCs in individuals with DSD in which the karyotypic and androgen-programmed transcript profiles are discordant. Based on our findings, it is possible that other transcript profiles in PBMC could be identified that correlate with sex or gender specific traits outside the genitalia. Were that the case, it might be possible to specify transcript signatures that reflect sex-specific differences in tissues such as the brain and in turn mirror sex-specific behavior, sexual orientation or gender identity. If so, subsets of the sex specific genes of our study could serve as future transcriptional biomarkers in DSD outcome studies in order to develop novel diagnostic tools contributing to decisions on gender assignment in DSD children.

## Conclusion

The sex chromosomes and the long term effects of the early embryonic androgen milieu as evidenced by the degree of genital masculinization in defined patients with DSD contribute independently to sex specific gene expression in PBMCs. This gives rise to 4 transcriptional categories of PBMC sexes: normal male, normal female and two "intersex" conditions with discordant alignments of the DSD individuals' genetic and hormonal sex signatures. This previously unknown architecture of sex specific gene expression in the human provides new perspectives for understanding the biology of sexual dimorphism in normal anatomy, physiology and behavior, its particular influences on diseases and its specific alterations in DSD.

## Methods

The study was approved by the ethical committee of the University of Lübeck, Germany.

### Normal controls and DSD patients

Blood samples were obtained from 34 individuals including 9 normal 46,XY males, 10 normal 46,XX females, one 46,XY male having CAH due to 21-hydroxylase deficiency and 14 individuals with DSD (Additional file 1). Mean age of normal males and normal females was 33 years, and 26.2 years, respectively (not different by t-test). Informed consent was obtained from control subjects, affected individuals, or their parents.

### RNA-isolation, RNA-amplification and aRNA-labeling

Blood samples were taken at 1:00 pm +/- 1 h to control for circadian influences on gene expression. White blood cell counts did not differ between normal males and females (by t-test). PBMC were isolated by Ficoll (Biochrom, Berlin, Germany) gradient centrifugation and total RNA was extracted by TRIZOL (Invitrogen, Karlsruhe, Germany). 3 μg of total RNA and 1 μg of T7-oligo(dT)15 were used for linear RNA-amplification. Reverse transcription was done by SuperScript II (400 U) (Invitrogen, Karlsruhe, Germany) at 42°C for 90 min. Second-strand synthesis buffer containing RNaseH (2 U), DNA-Polymerase-I (40 U), and dNTPs (1 mM) (Roche, Mannheim, Germany) was mixed with first reaction mixture and incubated for 2 h at 15°C. In-vitro-transcription-mixture containing T7-RNA-Polymerase (60 U) and NTPs (1 mM) (Roche) was added and incubated for 5 h at 37°C. 3 μg PBMC aRNA, 7.5 μg random-hexamer-primers (Roche), and Cy5-dUTP (0.1 mM) (Amersham, Freiburg, Germany) were used for Cy5-labeling, and 50 μg of human universal reference-RNA (Stratagene; Amsterdam; The Netherlands), 4.5 μg T20VN-primer (TIB MolBiol, Berlin, Germany), and Cy3-dUTP (0,1 mM) (Amersham) for Cy3-labeling.

### Microarrays and data analysis

DNA microarrays were obtained from Stanford Functional Genomics Facility , hybridized according to established procedures , scanned with GenePix4000B scanner, analyzed with GenePixPro 4.1 (Axon Instruments, Inc., Union City, CA) and loaded into Stanford Microarray Database [53]. Gene filtering, log_2 _transformation, microarray normalization and centering of fluorescence ratios were performed as described previously [11]. Genes with significant differences in transcription between normal males and females were identified by Significance Analysis of Microarrays (SAM) [20] and further analyzed by hierarchical clustering analyses [54]. The one 46,XY CAH individual was not part of the group of 9 normal males considered for SAM.

### Functional categorization of sex-specific genes

PANTHER was used to identify enrichment of biological processes with p-values < 0.05 considered as significant ([23]). GSEA was carried out with the Molecular Signature Database ([24]) using gene sets with at least 15 and maximal 500 genes. A p-value < 0.01 and a false discovery rate < 0.05 were considered significant.

### RT-PCR

Quantitative RT-PCR of XIST, DDX3Y and FZD6 was performed on two 46,XY male controls (N-003, N-015), two 46,XX female controls (N-020, N-031), two 46,XY DSD-females (P-019, P-033) and two virilized 46,XX CAH-individuals (P-103, P-105). Probes were FAM (F) labeled with dabcyl (DB) quencher. Exon-spanning primers and probes (TIB Molbiol, Berlin, Germany) had the following sequences: XIST-forward: ACCACCACACgTCAAgCTCTT; XIST-reverse: CTATCCTCAAgTgCTAgAgTgCCAg, XIST labeled probe: 6FAM-TTCCTACAAgCAgTgCAgAgAgCTgAgT-DB; DDX3Y-forward: gAAAAACAgAgTggAggAgCAAgTA; DDX3Y-reverse: gTCCACgATCATCAAATCTTCCC, DDX3Y labeled probe: 6FAM-ATAAAgACAgTTCAggTTggAgTTgCAgCA-DB; FZD6-forward: CgTCAgTACCATATCCCATgTCCTTA, FZD6-reverse: gAAATgACCTTCAgCTTgTgTgAAC, FZD6 labeled probe: F-AAAgCAAAAgCTCgACCAgAATTggCT-DB; TATA-binding protein (TBP) was used to normalize expression values (TBP-forward: CACgAACCACggCACTg-ATT, TBP-reverse: TTTTCTTgCTgCCAgTCTggAC; TBP-labeled probe: 6FAM-TgTgCAC-AggAgCCAAgAgTgAAgA-DB). 2 μg of total RNA, 0.5 μM dNTPs, 5 μM random decamers and 200 U of MMLV-RT polymerase were used per reverse transcription reaction. Heat denaturation for 3 min at 85°C was followed by reverse transcription for 1 hr at 44°C and subsequent inactivation for 10 min at 92°C. C-DNA corresponding to 12 ng of initial total RNA served as template. Primers (0.4 μM), probe (0.17 μM) and TaqMan Universal PCR mastermix (Applied Biosystems, Foster City, CA, USA) including Taq polymerase were added (final volume 25 μl). Initial denaturation at 95°C for 10 min was followed by 1 min cycling intervals at 60°C using RotorGene RG-3000 cycler (Corbett-Research, Sydney, Australia). Differential transcription was calculated by the ΔΔCT method [55].

## Abbreviations

DSD: disorders of sex development; CAH: congenital adrenal hyperplasia; SMD: Stanford Microarray Database; SAM: Significance Analysis of Microarrays; GSEA: Gene Set Enrichment Analysis; PANTHER: Protein ANalysis THrough Evolutionary Relationships.

## Authors' contributions

PMH was principal investigator, designed the study, performed clinical evaluation of DSD patients, microarray data analysis, and he was the leading writer. JHB was responsible for technical design of experiments, RNA-preparations, microarray hybridizations, database work, data analysis, and he was a writer. RW did genomic DNA sequence analysis in 46,XY DSD patients, RT-PCR, and was also a writer. JD is the leading database manager, performed data analysis and contributed to writing. ARU evaluated DSD patients, performed genomic DNA sequence analysis of CYP21A2, data interpretation and she was a co-writer. GC undertook the gene set enrichment analyses, data interpretation related to growth control and proliferation and wrote respective passages. MA contributed to microarray works and data analysis. RS performed data analysis and data interpretation. FR performed clinical evaluations of DSD patients and provided expert advice concerning genotype-phenotype correlation of 21-hydroxylase deficiency and other defects of steroid biosynthesis causing DSD. JDB is one of the senior writers and the senior advisor of microarray data interpretation, OH is senior writer, performed clinical evaluation of DSD patients, genomic DNA-sequence analysis of 46,XY DSD patients. He is senior advisor correlating clinical data, hormonal data and gene expression data. All authors read and approved the final manuscript.

## Supplementary Material

Additional file 1 **Phenotypic, clinical and molecular background information on the control individuals and the DSD patients corresponding to the PBMC sample numbers as indicated in the first column.** Click here for file

Additional file 2 **Text file to visualize data for Figure **1** together with additional files **3, 4** and **5** in TreeView **[54]. Click here for file

Additional file 3 **Zip file containing cdt file.** Cluster file to visualize data for Figure 1 together with additional files 2, 4 and 5 in TreeView [54]. Click here for file

Additional file 4 **Zip file containing atr file.** Array tree file to visualize data for Figure 1 together with additional files 2, 3 and 5 in TreeView[54] Click here for file

Additional file 5 **Zip file containing gtr file.** Gene tree file to visualize data for Figure 1 together with additional files 2, 3 and 4 in TreeView [54]. Click here for file

Additional file 6 **Text file to visualize data for Figure **3** together with additional files **7, 8** and **9** in TreeView **[54]. Click here for file

Additional file 7 **Zip file containing cdt file.** Cluster file to visualize data for Figure 3 together with additional files 6, 8 and 9 in TreeView [54]. Click here for file

Additional file 8 **Zip file containing atr file.** Array tree file to visualize data for Figure 3 together with additional files 6, 7 and 9 in TreeView [54]. Click here for file

Additional file 9 **Zip file containing a gtr file.** Gene tree file to visualize data for Figure 3 together with additional files 6, 7 and 8 in TreeView [54]. Click here for file
