# Supplementary material for: Disorders of sex development expose transcriptional autonomy of genetic sex and androgen-programmed hormonal sex in human blood leukocytes
Source: BMC Genomics. 2009 Jul 1;10:292. doi: 10.1186/1471-2164-10-292 (PMC2713997; doi:10.1186/1471-2164-10-292)
Supplement: Additional file 1 — Phenotypic, clinical and molecular background information on the control individuals and the DSD patients corresponding to the PBMC sample numbers as indicated in the first column. [file 1471-2164-10-292-S1.pdf]

Additional file 1

Phenotypic, clinical and molecular background information on the control individuals and the DSD patients corresponding to the PBMC sample numbers as indicated in the first column.

**PBMC sample**	**diagnosis**	**external genitalia *Prader stage***	**karyotype**	**age at time of blood sampling**	**gonadal status and hormonal therapy at time of blood sampling**	**clinical and molecular background**
**N-006**	female control	normal female	46, XX*	24;8	none reported	post pubertal
**N-009**	female control	normal female	46, XX*	31;5	none reported	post pubertal
**N-012**	female control	normal female	46, XX*	23;6	none reported	post pubertal
**N-020**	female control	normal female	46, XX*	35;6	none reported	post pubertal
**N-021**	female control	normal female	46, XX*	30;3	none reported	post pubertal
**N-029**	female control	normal female	46, XX*	8;3	none reported	pre pubertal
**N-031**	female control	normal female	46, XX*	35;6	none reported	post pubertal
**N-034**	female control	normal female	46, XX*	24;11	none reported	post pubertal
**N-036**	female control	normal female	46, XX*	24;5	none reported	post pubertal
**N-079**	female control	normal female	46, XX*	22;6	none reported	post pubertal
**N-003**	male control	normal male	46, XY*	40;4	none reported	post pubertal
**N-007**	male control	normal male	46, XY*	36;7	none reported	post pubertal
**N-008**	male control	normal male	46, XY*	25;8	none reported	post pubertal
**N-015**	male control	normal male	46, XY*	42;0	none reported	post pubertal
**N-016**	male control	normal male	46, XY*	42;0	none reported	post pubertal
**N-028**	male control	normal male	46, XY*	5;10	none reported	pre pubertal
**N-035**	male control	normal male	46, XY*	28;2	none reported	post pubertal
**N-080**	male control	normal male	46, XY*	33;2	none reported	post pubertal
**N-104**	male control	normal male	46, XY*	43;7	none reported	post pubertal, father of P-105
**P-004**	5α-reductase type II deficiency	P4	46,XY	20;4	gonadectomy at age of 2 yrs, *Cycloprogynova*	SRD5A2-mutation, homozygous, Arg111stop
**P-017**	17ß-hydroxysteroid-dehydrogenase type III deficiency	P2	46, XY	13;3	gonads in situ, no hormonal therapy at the time of blood sampling	HSD17B3-mutation, homozygous, donor splice site intron 3 position 325 plus 4

**P-018**	gonadal dysgenesis	normal female	46, XY	12;5	gonadectomy at age of 10;10 yrs (left side, seminoma) and 11;5 yrs (right side, seminoma), *Presomen 0.6mg*	normal SRY
**P-019**	gonadal dysgenesis	normal female	46, XY	16;11	gonads in situ, *Presomen 0.6 mg compositum*	normal SRY
**P-027**	17ß-hydroxysteroid-dehydrogenase type III deficiency	P2	46, XY	19;8	gonadectomy at age of 12 yrs, *Presomen 1.25 mg compositum Plus Presomen 0.3mg*	HSD17B3-mutation, Compound heterozygous, Asp130Ser, deletion of a T in codon 187 (downstream stop codon)
**P-032**	5α-reductase type II deficiency	normal female	46, XY	0;4	gonads in situ, no hormonal therapy at the time of blood sampling	SRD5A2-mutation, compound heterozygous, Arg108stop, insertion of an A in codon 165 (frameshift)
**P-033**	P450scc deficiency	normal female	46, XY	7;11	gonads in situ, hydrocortisone 3mg-1mg-1mg	CYP11A1-mutation, homozygous, deletion of an A in codon 279 (downstream stop codon)
**P-088**	mixed gonadal dysgenesis	P4	45, X0/ 46, XY	1;4	gonads in situ, no hormonal therapy at time of blood sampling	no mutational analyses
**P-089**	gonadal dysgenesis	normal female	46, XY	15;1	gonadectomy at the age of 14;8 yrs (dysgerminoma on left side), no hormonal therapy at time of blood sampling	no mutational analyses
**P-103**	21-hydroxylase deficiency, salt wasting CAH	P4	46, XX	9;3	gonads in situ, *hydrocortisone* 15mg/m^2 *fludrocortisone* 2 x 50μg	CYP21A2-mutation, homozygous, 8bp deletion in exon 3
**P-105**	21-hydroxylase deficiency, salt wasting CAH	P4	46, XX	10;2	gonads in situ, *hydrocortisone* 15mg/m^2 *fludrocortisone* 2 x 50μg	CYP21A2-mutations, compound heterozygous, Intron 2 splice, Arg356Trp
**P-110**	XY-DSD due to unknown defect of testosterone biosynthesis	P4	46, XY	2;11	gonads in situ, no hormonal therapy at the time of blood sampling	SF-1 normal, LH-receptor: heterozygous DNA- sequence alteration Val596Glu

**P-112**	21-hydroxylase deficiency, salt wasting CAH	P1	46, XX	14;5	gonads in situ, *hydrocortisone* 15mg/m^2 *fludrocortisone* 2 x 50µg, prenatal *dexamethasone* starting at 5th gestational week	CYP21A2-mutations, compound heterozygous, Arg356Trp, gene deletion
**P-113**	21-hydroxylase deficiency, salt wasting CAH	normal male	46, XY*	10;8	gonads in situ, *Hydrocortisone* 15mg/m^2 *fludrocortisone* 2 x 50µg	CYP21A2-mutations, compound heterozygous, Ile172Asn Intron 2 splice
**P-115**	XY-DSD due to unknown defect of testosterone biosynthesis	P1-2	46, XY	14;6	gonads in situ, no hormonal therapy at the time of blood sampling	no mutations in the following genes: androgen receptor, LH-receptor, CYP17

The degree of external genital virilization was classified according to Prader in both XX and XY DSD individuals to enable comparison of genital phenotypes independent of the sex chromosomes. Karyotypes marked with an "*" were initially deduced but not experimentally determined in the normal controls and in the one normal male CAH individual. They were later supported by sex-chromosome related expression patterns on microarrays (Fig 1).
